# *Genius Loci* of Ancient Village from the Perspective of Tourists Experience: Scale Development and Validation

**DOI:** 10.3390/ijerph19084817

**Published:** 2022-04-15

**Authors:** Zuoming Jiang, Derong Lin

**Affiliations:** School of Management, Xiamen University, Xiamen 361005, China; drlin65@xmu.edu.cn

**Keywords:** *genius loci*, modernity, ancient village, spiritual homeland, scale development

## Abstract

As the spiritual homeland marker of modern urbanites, ancient villages have grown into popular tourist attractions. However, the existing literature lacks a conceptually valid and psychometrically sound scale to measure the existential value of ancient villages that meet the material and spiritual needs of tourists. Guided by the concept of *genius loci* in architectural phenomenology, this study developed and validated a scale to measure the *genius loci* of ancient villages from the perspective of tourists experience in the Chinese context. Following multistage scale development and validation procedures, tourists in two World Cultural Heritage ancient villages were sampled in two stages (Study 1, *n* = 214; Study 2, *n* = 228) to establish the psychometric properties of a *genius loci* scale. A three-dimensional (earthbound atmosphere, architectural culture, and spiritual homeland) *genius loci* measurement with 10 items was identified, and the scale showed good reliability and validity. This study extends the current ancient village tourism literature and provides a measurement tool for further investigation by academics and tourism professionals.

## 1. Introduction

The emergence of mass tourism is the result of escaping modernity [1,2,3,4]. Modern urbanites have been looking for ideal tourism destinations [5,6,7]. Ancient villages, with the spatial attributes of ‘earthbound-culture’ have become popular destinations for urbanites to escape to [8,9]. Tourists expect to find the ideal lifestyle from the wisdom of traditional human settlements to help them better adapt to life and work [4,10]. In the early days of human history, ancient villages were the earliest concentrated settlements of people in European and Asian countries, which were mainly based on agricultural production. Ancient villages refer to villages with a long history of five or six hundred years, which are characterized by a complete cultural system and rich material and non-material cultural heritage [8]. The cultural system of ancient villages includes architecture, material culture, arts and crafts, rural customs and regional character [11]. From the perspective of tourists experience, ancient villages are sites for a kind of tourism that combines leisure with education [8]. Existing studies mostly focus on the surface application of cultural heritage protection and development [12,13], tourist experience and behaviour [14,15]. However, the driving forces behind the ancient village tourism boom are not clearly revealed, and the existence value of ancient village tourism has not been fully studied. To explore the essence of tourism, we need to return to the origin of ancient village as ‘home’. Ancient Roman philosophers and ancient Chinese sages have closely linked architecture with human survival and life, and endowed architecture with the significance of the ‘protective divinity of humankind’ [16,17]. Therefore, the exploration of the essence of ancient villages tourism can be inspired by the *genius loci* theory of phenomenology of architecture. This study introduces the theory of architectural phenomenology into the study of ancient village tourism, theoretically expanding the research fields of architecture, anthropology, and archaeology, and practically providing beneficial enlightenment for the cultural heritage management of ancient village.

*Genius loci*—the presiding spirit of a place—is defined as “the existential value of place that meet people’s material and spiritual needs is obtained through the interaction of human consciousness and action with local physical and cultural environment” [16]. From the perspective of tourists experience, the cognition of *genius loci* is a unique experiential quality that tourists feel as they blend in [18]. *Genius loci* conforms to tourists’ inner expectation of destination selection [18], and it is an important construct when exploring the essence of ancient village tourism from the perspective of the relationship between humans and architecture [16]. Thus, *genius loci* can further enrich the current knowledge of ancient village tourism research.

As an outstanding example of cultural heritages, ancient villages are favoured by tourists and have gradually become a research object for scholars. Numerous researchers have explored the tourist experience and behaviour in different countries, and studies generally show that ancient villages are destinations different from mass entertainment and leisure [8,14], which can provide tourists with strong ‘spiritual compensation’. This unique experience quality is the core element of the *genius loci* [19]. However, few studies have measured the *genius loci* of ancient villages. Specifically, core questions such as ‘What is the unique experiential quality that tourists get from ancient villages?’ and ‘How can this unique experiential quality be effectively measured?’ remain largely unanswered.

A possible reason for this deficiency is that the *genius loci* theory has only recently been introduced into tourism research, and the characteristics of ancient villages are not deeply understood. Tourism consumption behaviour, as a typical form of the combination of economy and culture, can be fully and properly explained when observed in the process of social development [4]. China is experiencing what Europe and North America experienced in the 1970s—the rapid development stage of urbanization. China’s urbanization rate was 63.89% in 2020 [20]. At the same time, traditional Chinese agricultural culture has been engendering a deep affection in people for rural areas for thousands of years [21]; ancient villages have become the homeland of traditional agricultural society, and the spiritual homeland of modern urban people. The dual ‘homeland identity’ is an ideal case for exploring the *genius loci* of ancient villages. Therefore, the findings of this study can not only verify the research conclusions of ancient village tourism in developed countries that have completed urbanization, but can also provide enlightenment in the development of ancient village tourism in developing countries that are still in the process of urbanization.

In recent years, researchers have explored the *genius loci* of a variety of destinations, such as a literary writer’s house [22], a seaside village in Cyprus [18], cultural landscapes of the Yoruba [23], and World Heritage sites of religious culture [24], confirming that the *genius loci* is a multidimensional construct with diversified characteristics. However, for the same type of destination experience, tourists can get a unique and universal feeling by integrating into it, which is exactly the *genius loci* dimension measured by researchers. The current measurement method is qualitative research, mainly through textual content analysis, to understand people’s subjective experience. The qualitative research paradigm lacks an effective measurement tool, which makes it difficult to compare the unique experiential qualities in different destinations [24], and it fails to provide a clear direction for the high-quality development of destinations [18]. Scale development is a rigorous quantitative procedure used to explore subjective and abstract constructs, which is conducive to an in-depth understanding of the connotation and structure of constructs [25,26]. To address this gap, a useful conceptual framework and a measurement scale for *genius loci,* needed to be constructed in the growing ancient village tourism market to enrich the ancient village tourism literature and provide practical management implications. Therefore, the research objectives are twofold: (1) identify the dimensions of the *genius loci* of ancient villages, and (2) develop and validate a *genius loci* scale (GLS).

## 2. Theoretical Background

### 2.1. The Ancient Village and the Genius Loci

**Figure 1:**
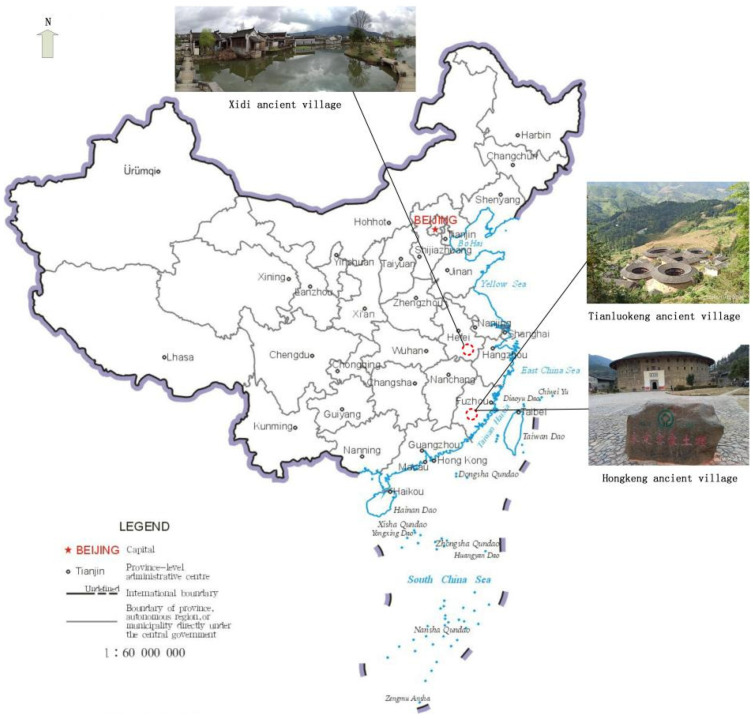
The ancient villages in Southern Anhui and Fujian Tulou.

*Genius loci* is an ancient concept coming from the Roman faith in a patron deity of places—that each kind of ‘independent’ noumena had its own genius that gave life to people and places, and determined their identity and essence [27]. Norberg-Schulz [28], a Norwegian architect, put forward the concept of *genius loci* based on Husserl’s phenomenology, Heidegger’s Dasein, Merleau-Ponty’s perception theory, and Piaget’s genetic epistemology. Norberg-Schulz believed that the generation of *genius loci* is the process of the long-term interactions between human subjective consciousness and the objective physical environment, in which people seek the authenticity of a place, form a personal identity of the place, and find its existential meaning [16]. The pursuit of *genius loci* represents people’s higher need for a true living space and the realization of self-value [18]. In recent years, *genius loci* have been explored in a variety of contexts. The *genius loci* of a literary writer’s house was identified as its domestic sphere, writer’s tools, and spirit of author using a qualitative analysis of 1200 Trip Advisor reviews [22]. Christou et al. [18] revealed that the *genius loci* of a seaside village in Cyprus includes physiological elements, social elements, and psycho-spiritual elements. Using an analysis of residents’ interview data, Silva [24] identified four interrelated *genius loci* dimensions of Bhaktapur, Nepal, as a World Heritage site of religious culture: a sense of sacredness, sense of community, sense of historicity, and sense of serenity. These studies have laid a solid foundation of theoretical contribution to the understanding and evaluation of *genius loci* of ancient villages. Following the development concept of harmony between humanity and nature, ancient villages have built characteristic dwellings according to local conditions, and have gradually formed cultural living spaces with local characteristics [29]. This study focuses on ancient villages in Southern Anhui and Fujian Tulou, which are typical representations of Chinese ancient villages. Among them, the former is characterized by Hui-style architecture and Huizhou culture, while the latter is exemplified by Tulou architecture and Hakka culture. The geographical location and architectural style of the two ancient villages are shown in Figure 1.

### 2.2. Theoretical Foundation of Genius Loci Dimensions of Ancient Villages

Norberg-Schulz [16] proposed three core elements of the *genius loci*: environment, culture, and belonging. Specifically, ‘environment’ refers to the atmosphere created by the human activities inside the building and the surrounding natural landscape; ‘culture’ refers to cultural symbols condensed in architectural materials, forms, and styles; and ‘belonging’ means to realise a poetic dwelling. The three core elements of environment, culture, and belonging are progressive and interlinked, providing a valuable analytical framework for the identification of the initial dimensions of this study.

Furthermore, according to the theory of place integration [30,31], the construction process of humans realising social meaning in a place occurs when people’s values, experiences, emotions, and sense of meaning are all fixed in the place context, which involves physical, emotional, and spiritual state changes [32,33]. The physical aspect is reflected in the place evaluation, which refers to people’s adaptation to the natural environment and local lifestyle; the emotional aspect is cultural adaptation, which means that people learn and identify with the local culture; the spiritual aspect is embodied in place attachment, which denotes the extent of people’s sense of belonging to place—of being ‘at home’. Tourists’ cognition of a destination’s *genius loci* moves from atmosphere evaluation to culture identification, which grows into place attachment. This provides a theoretical foundation for the confirmation of the three dimensions of the *genius loci* of ancient villages.

**Figure 2:**
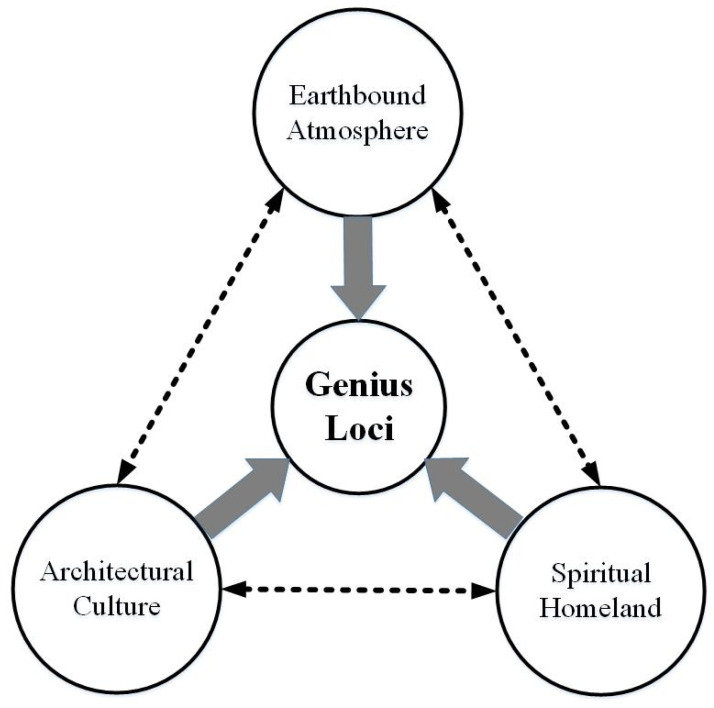
The conceptual framework of *genius loci* in an ancient village.

The ancient village tourism experience mainly involves appreciating the idyllic scenery, walking the ancient streets, tasting the local food, and experiencing ancient residential inns [14,15]. With the integration of experiential activities and the lengthening of trip durations, tourists establish an emotional connection with places, and find ancient village travel meaningful [34]. Gao and Wu [13] proposed that ancient villages have become the spiritual homeland of modern people by enhancing place identity and awakening cultural memory. According to the three-element structure of *genius loci* [16] and the theory of place integration [30,31], the unique experiential quality of ancient village tourism is comprehensive and mainly involves earthbound atmosphere (environment), architectural culture (culture) and spiritual homeland (belonging) [8,9,19]. Every aspect of unique experiential quality can lead to the formation of the *genius loci* of an ancient village. Therefore, earthbound atmosphere, architectural culture and spiritual homeland are the core dimensions of the *genius loci* of ancient villages. Accordingly, this study proposes a conceptual framework for the *genius loci* of the ancient village—a dynamic, two-way interaction system, as shown in Figure 2—which can be used to synthesise past research on tourist experience and behaviour in ancient villages (Table 1).

### 2.3. Potential Genius Loci Dimensions of Ancient Villages

#### 2.3.1. Earthbound Atmosphere of the Ancient Village

The first dimension is earthbound atmosphere, which can be understood as a sense of presence or place created by the human activities inside the dwellings and in the surrounding natural landscape—the day-to-day lifestyles of the village community, pastoral life, traditional agricultural practices, and traditional folk customs [8,14,34], not to mention the fresh air, stars at night, clear rivers, and idyllic scenery that help people feel they are returning to nature [14,15]. There are three indispensable conditions for the ‘ideal ancient village landscapes’ searched for by tourists: beautiful scenery, picturesque folk customs, and unique ethnic characteristics [8]. Many scholars [35,36] have suggested that earthbound atmosphere is a meaningful and valuable element, which is constructed by connecting the natural environment with human activities—that is, through the participation and interactive experience of tourists. Therefore, the earthbound atmosphere is an onsite experience of tourists blending into ancient villages.

#### 2.3.2. Architectural Culture of the Ancient Village

The second dimension is the architectural culture, which can be understood as the soul of a place. Architecture is the carrier of culture, which embodies a kind of aesthetic and humanistic charm [8]. The ancient village space as a material culture form includes the architectural space, street space, and overall space [37], which reflects the cultural symbol of ancient village architecture. Dayour et al. [9] and Zhang et al. [38,39] argued that architectural culture not only means that the ancient village meets some objective criteria (e.g., the harmony of humanity and nature, or the idea of geomantic omen), but more importantly, that tourists accept their socially constructed meaning and value (e.g., the ancient villages in Southern Anhui look like a painting; a Hakka ancient village is a safe place to live). Compared with the diversity of destination cultural landscape in previous studies [38,39], the architectural culture of ancient villages is a spatial order realized by the dwellings here, which can awaken cultural memory and enhance place identity for modern tourists [8].

#### 2.3.3. Spiritual Homeland of the Ancient Village

The third dimension is a spiritual homeland: a spiritual homeland refers to a place where people are emotionally attached [19]; ancient villages have become places that tourists yearn for and form an emotional attachment to. Researchers studying cultural tourism destinations have noted that a spiritual homeland is tourists’ most treasured emotional construct [19,40,41]. In this study, ancient villages have become the spiritual homeland that urbanites long for when traveling but cannot return to, and ancient village tourism can give urban tourists a feeling of homecoming, spurring a cultural roots-searching, and giving tourists the experience of ‘spiritual compensation’ [10,13].

**Table 1:** Supporting evidence sources of initial *genius loci* dimensions and items.

Initial Dimensions and Items	Supporting Evidence Sources
Earthbound Atmosphere	
GLS-1. I felt the heritage of the agriculture civilization	[18,42,43]; tourist interview; tourist message board
GLS-2. I felt the beautiful natural scenery	[24,43,44,45]; tourist interview; tourist message board
GLS-3. I felt the country life	[8,24,44]; tourist interview; tourist message board
GLS-4. I felt the slow life in the quiet environment of the ancient villages	[18,24,45]; tourist interview; tourist message board
Architectural Culture	
GLS-5. I enjoyed the harmony between people and nature	[8,42,44]; tourist message board
GLS-6. I enjoyed the philosophy of geomantic omens in the layout of ancient villages	[8,42,45]; tourist interview
GLS-7. I enjoyed the acquaintance society of traditional clans of people living together	[23,44,45]; tourist interview
GLS-8. The ancient dwellings everywhere make me feel the village’s rich history	[46]; tourist interview; tourist message board
Spiritual Homeland	
GLS-9. I found the spiritual homeland of my heart	[41,42,44,47]; tourist interview; tourist message board
GLS-10. I found a place to relax from the pressures of modern life	[41,42]; tourist interview; tourist message board
GLS-11. I found my ideal homeland to live in	[23,41,45,47]; tourist interview
GLS-12. I hope to return to and live in the ancient village after retirement	Tourist interview; tourist message board

## 3. Scale Development and Validation

### 3.1. Overview

As commonly identified guidelines by previous studies, a mixed method combining qualitative and quantitative surveys was recommended for scale development and validation [48,49,50,51]. First, the construct dimensions and scale items, grounded in the literature review, were generated, and there was an examination of tourist interviews, the tourist message board, and the expert panel. Second, a pilot study and Sample 1 data were used for scale purification. Third, Sample 2 data were used to assess the reliability and validity of the scale and develop norms for decision-makers. Table 2 provides an overview of the seven research phases, the results, and the destination survey rounds.

This study targeted Chinese ancient villages for three reasons. First, ancient villages in Southern Anhui and Fujian Tulou, two World Cultural Heritage Sites, have long been seen as China’s top ancient village destinations [13]. Second, as a ‘window display’ of traditional Chinese culture, the ancient village tourism industry of the two places is relatively mature, receiving millions of tourists per year, both domestic and international before COVID-19 [14]. Third, Chinese ancient villages are characterized by their wide distribution, multiple types, and cultural diversity. The ancient villages in Southern Anhui are mainly distributed in Huangshan city and Xuancheng city of Anhui Province, of which Xidi and Hongcun World Cultural Heritage sites in Yixian county are the most representative. The construction of ancient villages in southern Anhui is the result of the success of local people going out into business. In order to honor their family name, they built fine houses, ancestral halls, paved roads and bridges to make their hometown grand and imposing. Since the area was called Huizhou Fu in ancient times, historians have called this style of architecture the Hui-style. The Hui-style dwellings of ancient villages in Southern Anhui have horsehead walls and small green tiles, with exquisite carvings in stone, wood, and brick. Tulou ancient villages are mainly distributed in Longyan city and Zhangzhou city in the southwest of Fujian Province, with the World Cultural Heritage listed tulou houses in Yongding, Nanjing and Hua ’an counties as the most representative. Tulou houses were originally built to defend against foreign invasion during the wartime. During the Qing Dynasty and the Republic of China, local processing industries such as tobacco and tea flourished. As people became richer, they built larger tulou houses for their clan members to live together. This style is known as tulou architecture because its main building material is local clay. The Tulou dwellings in Fujian are large rammed earth buildings with tall and solid external walls, and round or square interior edges. Accordingly, the strong contrast of architectural features between the two cases can better satisfy the reliability and validity test of the GLS of an ancient village.

### 3.2. Study 1: Scale Development

#### 3.2.1. Specification of Construct Domains and Generation of Items

To specify the construct domains of *genius loci* to scale and create an initial pool of scale items [49], a series of qualitative research methods (literature review, tourist interview, tourist message board, and expert panel) were conducted from May to December 2020. First, the studies of *genius loci* in the architecture field provided the main framework for the development of the ancient village GLS. The ancient villages are distributed throughout the world—Italy, France, England, South Korea, the Philippines, etc. This study extracted and amended 11 related scale items (Table 1), then constructed three *genius loci* dimensions.

Second, onsite tourist interviews were conducted to collect information for extracting and verifying items and ensuring information saturation. To ensure that tourists have a deep understanding of the research topic, qualified interviewees must have spent at least one night in the ancient village, or be on at least their second visit to the village. A total of 22 tourists from the international youth hostel in the ancient village of Southern Anhui and the Fujian Tulou folk-dwelling inns were approached and interviewed. The number of interviewees was determined by the degree of information saturation, and the interview process ended when the interviewees had no further insights to share [52]. The length of the interview was 30–60 min. To help interviewees understand the subject better, the researchers explained the connotation of the *genius loci* of the ancient village. After receiving understandable feedback from interviewees, the in-depth interview was organized around the following open questions: (1) Could you share the special significance of choosing ancient villages as tourism destinations? (2) Could you share the general atmosphere of your ancient village tourism experience? (3) Could you talk about the ancient village elements that left a deep impression on you after your traveling? What meaning do these elements have for you? Tourists’ interviews were conducted and records of the responses were coded and analysed. Three potential *genius loci* constructs with 14 scale items were obtained through a three-step coding procedure.

**Figure 3:**
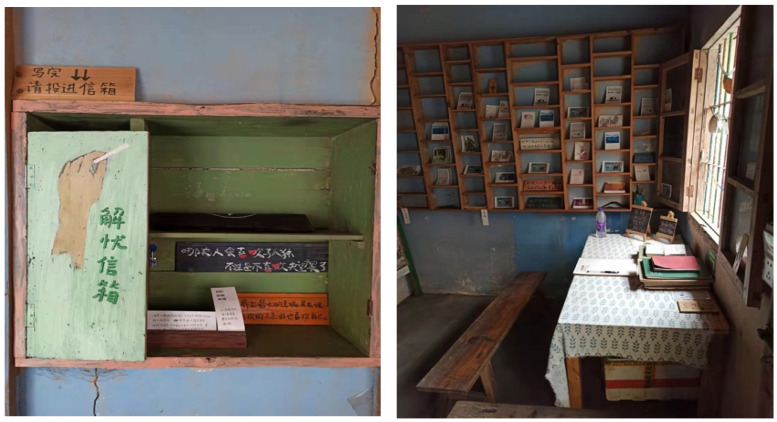
Message board in Xidi’s unattended grocery store (photograph by the first author). Note: On the left is a ‘remove sorrow’ mailbox.

Third, the message boards preserved in ancient villages contain personal stories, travel experiences, and inner thoughts of tourists in the ancient village. Compared with the quantitative research data of general formulas, tourist message boards are qualitative research texts with more case characteristics [53,54]. Some of the messages were banal, but in many cases the message board communications were very deep, and some tourists wrote about their emotional engagement in interacting with the ancient villages. The research team came across an unattended grocery store in Xidi ancient village, which not only sold a few groceries but also had a ‘remove sorrow’ mailbox on the wall, containing 16 tourist messages written between September 2017 and September 2020 (Figure 3). First, we scanned 16 tourist message boards page by page, obtaining a total of 1178 scanned pages of photos, deleting messages that had little relevant content. After screening, a total of 254 relevant messages were obtained, for a total of 38,718 words. Based on previous studies of tourist message board content [55,56], a three-step manual coding and thematic analysis of the tourist message board content helped verify three *genius loci* constructs with 12 related scale items [57]. By incorporating the results, an initial pool of 18 scale items was generated.

Fourth, to evaluate the scale’s content validity, the constructs and scale items were sent to seven experts for comments and suggestions in December 2020. Five are university scholars, and have rich experience in ancient village field investigation; the other two are managers of ancient village tourism development enterprises in Huangshan City, Anhui Province. They reviewed and rated the representativeness and rationality of the scale items, with 1 as strongly inapplicable and 5 as strongly applicable. The experts could also provide comments and suggestions. Finally, according to feedback from the experts, six initial items with low ratings were deleted (‘I felt the affinity of the residents’; ‘I felt peace of mind’; I had a feeling of cultural root-searching for ancient villages’; ‘I found a harmonious coexistence between humanity and nature’; ‘I agree with the current development of ancient villages’; and ‘I enjoyed the balance of history and culture with modern life’), and the statement of each item was modified and finalized. A total of 12 questionnaire items were finalized (Table 1).

#### 3.2.2. Pilot Study

The purpose of the pre-survey was to evaluate the quality of the initial questionnaire, further revise the initial items, and obtain the questionnaire for the formal survey. Data were collected in Tianluokeng, a Fujian Tulou ancient village, with 100 questionnaires distributed by convenience sampling; a total of 92 valid questionnaires were collected. Cronbach’s alpha and item-to-total correlations were calculated to assess the reliability of the whole scale and each item [49]. As a result, the alpha scores were higher than 0.7, and item-to-total correlations were higher than 0.4, which indicated that the structure of the scale was reliable and valid [58]. A total of 12 items remained.

#### 3.2.3. Data Collection

The first round of data collection was conducted in the Xidi ancient village. The questionnaire contained sociodemographic information and trip characteristics of the respondents, followed by the 12 GLS items, using a seven-point Likert-type scale (1 = completely disagree, 7 = completely agree).

The field survey approached potential respondents in the international youth hostels and the public leisure areas of the Xidi ancient village. The screening requirements of qualified respondents were the same as those of tourist interviews—that is, they must have had at least a second trip or a one-night stay. Eventually, a total of 260 (214 usable) responses were collected through convenience sampling, with an effective rate of 82.31%. The subject-to-item ratio was 17.83:1, which was higher than the limited criteria of 5:1 [59]. The absolute values of univariate skewness did not exceed 2 while the absolute values of univariate kurtosis did not exceed 3, which indicated that the data did not appear to deviate ‘extremely’ from a normal distribution [60].

The demographic and trip characteristics of the respondents are shown in Table 3. The urban respondents outnumbered their country counterparts (52.3% vs. 47.7%). Male respondents (53.8%) slightly outnumbered their female counterparts (46.2%). The top three occupations were students (25.5%), managers of enterprise (21.2%), and teachers (16.0%). Most respondents were young or middle-aged; those in the 18–35 age range accounted for 55.4%, with ages 36–45 accounting for 20.7%. Most (51.4%) held an undergraduate degree. In terms of income, 27.2% earned less than ¥3000, while 26.2% earned more than ¥10,001. Moreover, the respondents’ trip characteristics are also shown in Table 2. The length of stay of most respondents were one day (43.2%) and two days (40.8%). Among the visitors to Xidi ancient village, 53.1% of the respondents were visiting for the first time, 24.9% for the second time, 12.2% for the third time, and 9.4% for the fourth time and above.

#### 3.2.4. Purification of Measures

An exploratory factor analysis (EFA) was conducted to determine the dimensions of the scale items, using principal component analysis with maximum variance rotation. The following principles are followed to extract common factors: first, the eigenvalue is greater than or equal to 1; second, the item is deleted when the factor load after rotation is less than 0.5 or more than 0.4 on multiple factors at the same time [58]. In the first round of EFA (KMO = 0.847; Bartlett’s test of sphericity = 750.111; df = 66; *p* < 0.001), the item GLS-5 was deleted due to low communality (<0.5) and low factor loading (<0.5). The second round of EFA (KMO = 0.825; Bartlett’s test of sphericity = 631.482; df = 55; *p* < 0.001) was conducted following the same procedure with the 11 remaining items. Eventually, the three factors’ latent structure identified with the remaining 11 items, explaining 60.494% of the total variance (Table 4), demonstrating relatively high representativeness [61]. The alpha of all three factors were above 0.7, indicating that the data quality was reliable [58]. The factor structure is consistent with the theoretical analysis, indicating that the results have a reasonable theoretical basis.

### 3.3. Study 2: Scale Validation

#### 3.3.1. Data Collection

To further validate the GLS, a second survey round was conducted. The questionnaire contained three parts. The first part was the same question about sociodemographic information and trip characteristics of the respondents as in study 1. The second part consisted of the retained 11 GLS items. In the third part, in order to further verify the criterion-related validity of the GLS, the relationship between *genius loci* and a related mature construct needed to be examined [62]. According to the previous studies [18,34], *genius loci* is a significant inner need whose generation and development are accompanied by the existential value of ancient villages as recognized by tourists. This recognition may lead to the improvement of onsite stickiness and post-travel stickiness of tourists in their destination tourism intention [41,63]. Accordingly, there is a high potential correlation between *genius loci* and tourist stickiness. One of the classic social psychological theories in tourism research—cognitive–affection–intention theory [64]—explains this relationship: if tourists have a higher ancient village *genius loci* recognition, it will arouse their emotional resonance and positively affect their tourism intention. Hence, tourist stickiness meets the requirements of the criterion variable selection [62]. Accordingly, the third part of the study adopted the revised tourist stickiness scale of Gao et al. [63] and Hung et al. [41]. The second and third parts used a seven-point Likert scale (1 = completely disagree, 7 = completely agree).

The second round of data was collected in Hongkeng ancient village, in folk-dwelling inns and public leisure areas. The same survey administration procedure as reported in Study 1 was applied in Study 2. A total of 280 (228 usable) responses were collected through convenience sampling, with an effective rate of 81.43%. The subject-to-item ratio was 20.73:1, which was higher than the limited criteria of 5:1 [59]. For 11 scale items shown, the data did not appear to deviate ‘extremely’ from a normal distribution [60]. As shown in Table 3, the profiles and trip characteristics of Sample 2 were mostly similar to those in Sample 1.

#### 3.3.2. Assessment of Reliability and Validity

Given that all scale constructs were measured by self-management reports, common method bias (CMB) was a serious concern. To examine CMB, we conducted Harman’s single-factor analysis, one of the most widely utilized techniques for testing its potential threat [65]. Three factors with eigenvalues greater than 1 emerged; the total variance of a single factor was 39.141% (<50%). These results indicate that the data were unbiased against CMB [65].

The reliability and validity of the latent structure were measured by confirmatory factor analysis (CFA). The fit indexes of the initial measurement model showed that one index did not meet the acceptance standard (χ^2^/df = 2.363, GFI = 0.933, IFI = 0.930, CFI = 0.929, NFI = 0.885, TLI = 0.905, and RMSEA = 0.077). Following Anderson and Gerbing [66], we deleted item GLS-4, which had low factor loading values, to improve the goodness of fit. The fit indexes result of the new measurement model structure were χ^2^/df = 2.032, GFI = 0.950, IFI = 0.953, CFI = 0.952, NFI = 0.912, TLI = 0.933, and RMSEA = 0.067. Altogether, these values of fit indexes indicated a good fit of the three-factor model to Sample 2 data [58].

The next step was to examine the reliability and validity of GLS. As shown in Table 5, the factor loadings reached significant levels for all items greater than 0.5 (0.566–0.829). The individual item reliability (R^2^) of all structures was greater than 0.3 (0.319–0.688). The composite reliability (CR) of the model was greater than 0.7 (0.7796–0.8142). The t values for 10 items were between 8.057 and 13.769 (*p* < 0.001). The average variance extracted (AVE) values were between 0.5271 and 0.5517. According to Fornell and Larcker [67], the convergent validity of the construct is still adequate, and the convergent validity of the GLS was confirmed. Table 6 shows that the square roots of AVE of all dimensions were greater than the correlation coefficient among all dimensions, indicating that the scale construct had good discriminant validity [58]. Thus, the assessment of the measurement model strongly supports the reliability and validity of the latent constructs.

#### 3.3.3. Criterion-Related Validity

As mentioned above, this study conducted a criterion-related validity test to further evaluate the validity of the GLS. As shown in Table 7, there was a significant and positive correlation between three *genius loci* dimensions, the overall *genius loci*, and two tourist stickiness intentions (i.e., onsite stickiness and post-travel stickiness). In terms of correlation coefficient, the overall *genius loci* had the greatest positive impact on the two tourist stickiness intentions, followed by the spiritual homeland dimension, while the earthbound atmosphere and architectural culture dimensions have the least impact. Therefore, these results provide sufficient evidence of criterion-related validity for the GLS.

A dimensionality test was next conducted to confirm whether the three-factor model was the more appropriate conceptualization of *genius loci*. Specifically, a one-factor model (i.e., all items of the three GLS components loading on one factor) was estimated. As displayed in Table 8, the CFA fitting results of the one-factor model were significantly worse than the three-factor model (∆χ^2^(3) = 140.790, *p* < 0.001). Similarly, a two-factor model (i.e., combining architectural culture and spiritual homeland, the two most highly correlated components, while leaving the earthbound atmosphere factor unchanged) was estimated, and the two-factor model results exhibited a significantly worse fit than the three-factor model (∆χ^2^(2) = 65.011, *p* < 0.001). These results further support the proposed three-factor model.

## 4. Discussion and Conclusions

Following a rigorous step in previous studies on scale development and validation, the study established the validity and reliability of the GLS through two successive studies. Specifically, using EFA, with the three *genius loci* dimensions, 11 items were retained in the identified scale. The construct dimension and measurement scale were further verified through CFA. Along with the satisfactory reliability and validity, statistical results also demonstrated adequate criterion-related validity of the GLS. The factor analysis results showed that architectural culture and earthbound atmosphere factors should be optimized toward tourists’ satisfaction. Therefore, the development and validation of the GLS has significant theoretical and practical implications.

### 4.1. Theoretical Implications

This present study was the first to introduce the *genius loci* theory of phenomenology of architecture into ancient village tourism, and it provides a new theoretical perspective for exploring the essence of ancient village tourism. The concept of *genius loci* can effectively establish the relationship between tourists and ancient villages, which can push the existing surface application research of ancient village tourism to a greater depth exploration [16,18,19]. This study identified and confirmed that the three elements of earthbound atmosphere, architectural culture and spiritual homeland together constitute the significant dimensions of the ancient village *genius loci*, enriching the growing body of literature on this topic. The three-dimension measurement scale provides a powerful research tool for subsequent studies to evaluate how tourists—even in different destination contexts—reflect on the important question, of ‘Where am I?’ and ‘What is the unique experiential quality that tourists get from a destination?’

As indicated in Table 4, the eigenvalue and the explained variance show that the spiritual homeland is the most prominent component of ancient village *genius loci*, followed by architectural culture and earthbound atmosphere. Against the backdrop of rapid urbanization, the sense of homecoming and cultural root-searching are becoming powerful driving forces for modern urbanites travelling to ancient villages [68]. Ancient village tourism behaviour is a window through which modernity can be interpreted [69,70].

It is noteworthy that architectural culture was identified as a second prominent component of ancient villages’ *genius loci*. This finding suggested that most tourists find cultural resonance in the ancient village tourism experience, in ‘the wisdom of traditional human settlement culture’ [71,72]. Urban tourists not only visit ancient villages to relieve homesickness, but also to help their children learn about ancient village history and culture [14,15]. To sum up, a salient cultural identity to the ancient village may exist.

Earthbound atmosphere was identified as the least important component of ancient village *genius loci*, due perhaps to the commercialization of ancient village tourism [73,74]. Sun et al. [75] proposed three factors of tourism commercialization—social, business, and commodification circumstances. Most residents of the three ancient villages in this study are engaged in tourism catering, shopping, and accommodation services, while few are engaged in agricultural production. The seasonal agricultural cultural landscape (rapeseed flowers and rice) was planted for tourists to enjoy the scenery at the expense of local governments. Therefore, commercialization reduces the agricultural emphasis, aggravates the noise in the ancient streets, and negatively affects the earthbound atmosphere of the ancient villages to a certain extent.

**Figure 4:**
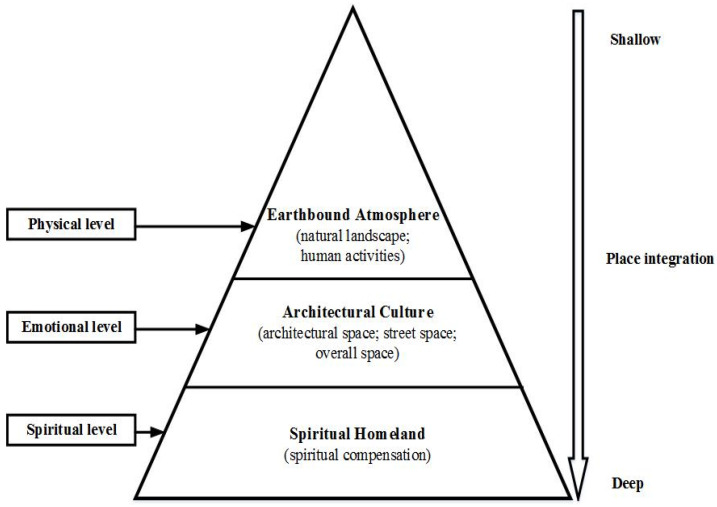
The model of *genius loci* in an ancient village.

The current study provides a theoretical model with empirical evidence to confirm that the theory of place integration can explain the structure formation process of the *genius loci* of ancient village, thus expanding the theoretical contribution of place integration to the study of the *genius loci*. Figure 4 shows the pyramid structure of the *genius loci* of ancient villages, which unfolds from top to bottom on three levels: physical, emotional, and spiritual. When tourists came to an ancient village, the first stimulus is the natural environment and local lifestyle. Then, tourists learn about local history and adapt to local culture in various architectural spaces, and establish an emotional connection with the place [14,15]. Finally, when tourists blend into local life and find an ideal lifestyle, the significance of ancient villages as ‘spiritual homeland’ is realised [13,34]. Therefore, tourists’ *genius loci* cognition of ancient village is an integration process from shallow to deep, from accumulation to qualitative change.

Finally, as shown in Table 7, the three *genius loci* components, and the GLS as a whole, were significantly and positively related to onsite stickiness and post-travel stickiness. In other words, with a strong *genius loci* recognition, tourists will have high intentions of onsite stickiness and post-travel stickiness. These relationships are consistent with the previous literature [41,42,63]. Considering the close relationships among tourism intention, motivation, satisfaction, and identity [76,77], the effect of *genius loci* on tourist perception and behaviour needs to be strengthened.

### 4.2. Practical Implications

This study provides practical implications for destination managers, tourism policy-makers, and tourism marketers to better understand tourists’ *genius loci* of ancient villages by using the findings of this research. First, this study shows that spiritual homeland and earthbound atmosphere were the most and least important poles, respectively, of the ancient village *genius loci*, which reflected the dilemma of tourists’ experience: tourists loved ancient villages and sensed a spiritual homeland, but were not satisfied with the commercialized atmosphere of ancient village tourism. Therefore, the destination managers could take the *genius loci* dimensions as an important monitoring index for the sustainable development of ancient village tourism, with real-time monitoring of the negative effects of cultural commercialization, resource capitalization, and consumption climbing. Second, to strengthen the *genius loci*, efforts should focus on enhancing the internal psychological benefits of tourists. Tourism policy-makers should consider ways to satisfy tourists’ homesickness by creating atmosphere, highlighting local cuisine, providing products and services, etc. Third, tourism marketers could emphasize the ancient village *genius loci* that could meet tourists’ material and spiritual needs to attract potential tourists. Precision marketing could differentiate ancient village tourism from mass tourism products to attract visitors.

## 5. Limitations and Future Research

Several limitations of this study should be acknowledged. First, as this study was based on two Chinese ancient village samples, the findings may not be generalizable to tourists from other countries visiting other ancient village destinations. Given the urbanization stage, history, and cultural differences of different countries, the dimensional structure and items of the *genius loci* require further verification and development in countries that have completed or are undergoing urbanization. Additional research is needed to test the validity of the scale in developed countries such as Europe and America and developing countries such as Asia and Africa. Second, the conceptual framework of *genius loci* is a dynamic, two-way interaction system; however, this study did not focus on their temporal dynamics and interaction relationships. We will discuss these intrinsic laws in a follow-up study to enrich the theoretical system of *genius loci* constructs. Beyond the above-noted limitations, future research is encouraged to apply the GLS in examining the antecedents and consequences of *genius loci*.

## Figures and Tables

**Table 2:** Overview of the research process phases.

Research Process	Objectives	Results	Destination Survey
Phase 1: Literature Review	Identify *genius loci* dimensions	three *genius loci* dimensions	
Phase 2: Interviews	Identify *genius loci* indicators	22 tourists’ interview three *genius loci* dimensions 14 indicators identified	Ancient villages in Southern Anhui: Xidi, 5–7 September 2020 (three days) Fujian Tulou ancient village: Tianluokeng, 26–29 November 2020 (four days)
Phase 3: Message Board	Identify *genius loci* indicators	16 tourists’ message board three *genius loci* dimensions 18 indicators identified	Ancient villages in Southern Anhui: Xidi, 5–7 September 2020 (three days)
Phase 4: Expert Panel	Find consensus between *genius loci* dimensions and indicators	seven experts three *genius loci* dimensions accepted six indicators removed 12 indicators identified	
Phase 5: Pilot Study	Measure purification	92 valid questionnaires three *genius loci* dimensions 12 indicators identified	Fujian Tulou ancient village: Tianluokeng, 1–8 January 2021 (eight days)
Phase 6: Measurement Scale Development	Explore dimensionality	214 valid questionnaires three *genius loci* dimensions one indicator removed Validation measurement instrument	Ancient villages in Southern Anhui: Xidi, 11–19 March 2021 (nine days)
Phase 7: Measurement Scale Validation	Assessing reliability and validity Criterion-related validity	228 valid questionnaires Confirmed three *genius loci* dimensions one indicator removed Nomological validity supported	Fujian Tulou ancient village: Hongkeng, 2–9 May 2021 (eight days)

**Table 3:** Demographic profiles and trip characteristics of respondents.

Variable	Category	Distribution, *n* (%) (*n* = 214)	Distribution, *n* (%) (*n* = 228)
Birthplace	Country	102 (47.7%)	133 (58.3%)
	Urban	112 (52.3%)	95 (41.7%)
Gender	Male	114 (53.8%)	75 (32.9%)
	Female	98 (46.2%)	153 (67.1%)
Occupation	Government staff	19 (9.0%)	17 (7.5%)
	Manager of enterprise	45 (21.2%)	23 (10.1%)
	Worker	3 (1.4%)	2 (0.9%)
	Service staff	6 (2.8%)	8 (3.5%)
	Peasant	3 (1.4%)	1 (0.4%)
	Doctor	2 (0.9%)	2 (0.9%)
	Teacher	34 (16.0%)	49 (21.5%)
	Retired	12 (5.7%)	3 (1.3%)
	Student	54 (25.5%)	102 (44.7%)
	Freelancer	14 (6.6%)	8 (3.5%)
	Other	20 (9.4%)	13 (5.7%)
Age	<18	4 (1.9%)	3 (1.3%)
	18–35	118 (55.4%)	163 (71.5%)
	36–45	44 (20.7%)	35 (15.4%)
	46–60	33 (15.5%)	22 (9.6%)
	>60	14 (6.6%)	5 (2.2%)
Education	Junior high school and below	7 (3.3%)	4 (1.8%)
	Senior high school	15 (7.1%)	6 (2.6%)
	Junior college	29 (13.7%)	19 (8.3%)
	Undergraduate	109 (51.4%)	125 (54.8%)
	Postgraduate or above	52 (24.5%)	74 (32.5%)
Personal Monthly Income (RMB)	≤3000	56 (27.2%)	97 (42.5%)
	3001–5000	24 (11.7%)	36 (15.8%)
	5001–7000	31 (15.0%)	28 (12.3%)
	7001–10,000	41 (19.9%)	37 (16.2%)
	≥10,001	54 (26.2%)	30 (13.2%)
Length of Stay in Ancient Village	One day	92 (43.2%)	98 (43.0%)
	Two days	87 (40.8%)	73 (32.0%)
	Three days	19 (8.9%)	24 (10.5%)
	≥Four days	15 (7.0%)	33 (14.5%)
Number of Visits to Ancient Villages	First time	113 (53.1%)	110 (48.2%)
	Second time	53 (24.9%)	52 (22.8%)
	Third time	26 (12.2%)	32 (14.0%)
	Fourth and above time	20 (9.4%)	34 (14.9%)

**Table 4:** Exploratory factor analysis results (sample 1, *n* = 214).

Factor/Item	Factor Loading	Eigenvalue	Variance Explained (%)	Cumulative Variance Explained (%)	Cronbach’s α
Spiritual Homeland		3.987	36.246	36.246	0.741
GLS-11	0.767				
GLS-9	0.733				
GLS-12	0.707				
GLS-10	0.683				
Architectural Culture		1.427	13.974	50.220	0.702
GLS-8	0.754				
GLS-7	0.749				
GLS-6	0.617				
GLS-4	0.575				
Earthbound Atmosphere		1.020	10.274	60.494	0.704
GLS-2	0.787				
GLS-1	0.750				
GLS-3	0.730				

**Table 5:** Confirmatory factor analysis results (Sample 2, *n* = 228).

Factor/Item	Factor Loading	*t*-Value (C.R.)	SMC (R^2^)	Composite Reliability (CR)	Average Variance Extracted (AVE)
Spiritual Homeland				0.8142	0.5271
GLS-11	0.817	13.769	0.668		
GLS-9	0.721	11.655	0.520		
GLS-12	0.566	8.596	0.320		
GLS-10	0.775	12.821	0.600		
Architectural Culture				0.7796	0.5415
GLS-8	0.758	9.528	0.433		
GLS-7	0.751	10.975	0.564		
GLS-6	0.697	8.549	0.357		
Earthbound Atmosphere				0.7855	0.5517
GLS-2	0.725	8.970	0.391		
GLS-1	0.829	11.863	0.688		
GLS-3	0.665	8.057	0.319		

**Table 6:** Construct correlations and squared roots of AVE (Sample 2, *n* = 228).

	Earthbound Atmosphere	Architectural Culture	Spiritual Homeland
Earthbound Atmosphere	0.6824		
Architectural Culture	0.517	0.6717	
Spiritual Homeland	0.497	0.610	0.7260

Note: The diagonal elements are the squared roots of AVE; the off-diagonal elements are the correlations between the constructs (*p* < 0.05).

**Table 7:** Path analysis results for *genius loci* subscales predicting stickiness intention (Sample 2, *n* = 228).

	Earthbound Atmosphere	Architectural Culture	Spiritual Homeland	Overall *Genius Loci*
Onsite Stickiness	0.500 **	0.492 **	0.516 **	0.645 **
Post-travel Stickiness	0.437 **	0.414 **	0.545 **	0.608 **

Note: ** *p* < 0.01.

**Table 8:** Model comparisons for dimensionality (Sample 2, *n* = 228).

	CMIN	df	CMIN/df	*p*-Value	GFI	NFI	TLI	CFI	IFI	RMSEA
One-factor Model	205.830	35	5.881	0.000	0.838	0.721	0.683	0.753	0.757	0.147
Two-factor Model	130.051	34	3.825	0.000	0.896	0.824	0.816	0.861	0.863	0.112
Three-factor Model	65.040	32	2.032	0.000	0.950	0.912	0.933	0.952	0.953	0.067

## Data Availability

The dataset used in this research are available upon request from the corresponding author.
